# The effect of psychrometry on the performance of a solar collector

**DOI:** 10.1007/s11356-021-16353-5

**Published:** 2021-09-30

**Authors:** Alok Dhaundiyal, Gedion H. Gebremicheal

**Affiliations:** Institute of Process Engineering, Szent Istvan University, Godollo, Hungary

**Keywords:** Exergy, Heat transfer, Psychrometry, Enthalpy, Solar radiation, Collector plate

## Abstract

The investigation of a solar collector is based on the thermal behaviour of a carrier fluid and the degradation of energy across a flat plate collector. The exergy analysis of a thermal system includes the change in the exergy function of a carrier fluid while transferring solar radiation across an air gap. The cell cast acrylic glass was used to transmit the incident solar radiation to the absorber plate, and to safeguard the absorber plate from the outside environment. With the help of the steady flow energy equation, the enthalpy of the carrier fluid (moist air) was calculated. The specific humidity of the incoming air was calculated at an average dry bulb temperature of 299.4 K. The stagnation temperature at a limiting condition was also estimated to find out the maximum permissible limit for a given thermal design. The mass flow rate of air was assumed to be 5.2 g-s^−1^. The efficiency of the solar collector was found to vary from 40 to 42%, whereas the thermal energy available for drying was 15–59% of the exergy of the carrier fluid. The net entropy generation rate due to the collector plate was calculated to be 0.12 W-K^−1^.

**Supplementary Information:**

The online version contains supplementary material available at 10.1007/s11356-021-16353-5.

## Introduction

The conversion of terrestrial radiation into thermal energy is one of the functions of a solar thermal system. The application of these systems is extensively making its grip in the various branches of the renewable energy field. As compared to other renewable energy systems, they have relatively less robustness and complexity in their structure, yet a query has been raised about their effectiveness during the transformation of one form of energy into another. Since geographical diversity might cause deviation in the output of the solar collector, therefore, it is essential to examine those intensive properties of the medium that might influence the energetic aspect of a solar system. In the same context, one of such solar systems, solar collector, using in the post-harvesting application has been critically examined based on thermodynamic and heat transfer principles, which are backed up by psychrometric detailing of the conditioned carrier fluid to measure the qualitative edge of the given system.

Some studies were conducted to bring a structural reform in the existing solar collector system (Jain et al., 2017; Dhaundiyal et al., 2021; Dhaundiyal and Atsu, 2021; Pramanik et al., 2017). Yeh (1994) had noticed that fins and baffles on the absorber plate were an efficient way of improving the heat transfer abilities of a solar air heater and causing turbulence. It was concluded that the increasing rate of flow of mass enhances the solar collector efficiency. Another factor was the extended area, increasing baffle in each fin was found to influence the overall performance of a solar collector (Yeh, 1994). But with this modification, it was estimated that the overall production cost of an absorber plate would increase to 4.5–150 US$-kg^−1^(Tiris et al., 1995). So, quantitative analysis was found to have a trade-off relationship with the cost of production of a collector system (Tiris et al., 1995). In addition to fins and baffles, it was observed that introducing the artificial roughness on the absorber plate can improve the performance of the solar collector (Prasad, 2013; Karwa and Chitoshiya, 2013). In one of the experiments, the thin G-I wires having varying diameters (*e*/*D* = 0.0092−0.0279) were embedded in the absorber plate along the course of a carrier fluid, and it was found that inertial force of the carrier fluid alongside with relative height of roughness at a particular relative roughness pitch influenced the thermal efficiency of the solar collector. It was reported that the introduction of artificial roughness in the solar air heaters relatively increased the overall heat transfer as compared to the smooth solar collector working under similar operating conditions. It was found that the Nusselt number increased from 45 to 54 as the relative roughness of the surface varied from 0.011 to 0.027 over the smooth surface. However, it was noticed that the change in Nusselt number was ramped faster for rough surface than smooth one when Reynold number was increased to 10,000 (Prasad, 2013). The reason is quite clear that the change in surface roughness influences the boundary layer flow and the contact area between fluid and wall, and thus the heat transfer is varied. However, the change in roughness also impacts the friction losses encountered during the flow. It was reported that the twisted square duct and elliptical pipes could be used to enhance the heat transfer across the passage. Upon investigating the heat transfer characteristic for the broad range of *Re* and *Pr* along the twisted course, it was found that the swirl and secondary flow was developed at the corner which ultimately led to enhance the heat transfer coefficient (Bhadouriya et al., 2015). The baffles are often used as a mean of creating artificial roughness to develop turbulence in the air channel and enhance the heat transfer rate. The type of generated geometry on the surface also has a different effect on the heat transfer rate. It was noticed that velocity magnitude near the wall surface was higher for rounded transition to the flat surface than that of a conventional ribbed surface which consequently influences the heat transfer rate. It was seen that the generated dimple on the absorber side had a maximum thermal performance factor of 1.4 for relative roughness height of 0.036, relative roughness pitch of 10 and arc angle of 60° (Sethi et al., 2012). These heat transfer enhancement schemes, rough or extended surfaces, reduce the thermal resistance either by influencing the effective heat transfer surface area or by creating turbulence in the fluid flowing across the passage.

From a different point of view, Goering et al. (1997) explained numerically with the help of Navier Stokes momentum and energy equations that the Dean number (*De*) and buoyancy are the prime factors to influence the friction factor ratio or vice versa. As the Grashof number (*Gr*) increases, the geometry of the surface plays a diminutive role. Honestly speaking, friction factor is a subsidiary parameter to enhance the heat transfer across the fluid boundary since the heat transfer characteristics are not solely rely on velocity profile but also the function of the temperature field. The gradient of streamwise velocity and temperature both vary at boundary if *Gr* and *De* are altered. Suffice to say, the heat transfer mechanism can also be influenced by some other underlying factors and not only by the surface characteristic.

Similarly, the packed bed materials were filled between the absorber and glass cover and allowing the heat to transfer to the air through convection. The overall thermal output was increased for a stipulated time, but the increase in the overall heat transfer coefficient was not found to be significantly high (Karwa and Chitoshiya, 2013). Some other structural modifications in the solar collector system were also carried out by using corrugate surfaces such as V-corrugate and cross-corrugate surfaces (Ramadan et al., 2007; Dović and Andrassy, 2012) and recirculation of the flowing air in differently configured solar collectors (Ho et al., 2005a; Ho et al., 2005b). There was also another combination where the absorber was replaced by a wire mesh. Each air channel had seven steel mesh layers (4 mm^2^), which were painted black before installing into the system. It was reported that the thermal efficiency of the solar collector would increase if the number of air pass increased (Omojaro and Aldabbagh, 2010), but the structural effect of the wire mesh on the thermal solar collector system was omitted. In another work, Krishnananth and Kalidasa (2013) stated that the performance of a thermal system could be improved by integrating the phase-change material with a double-pass solar air heater. They concluded that the temperature gradient had the least deviation throughout the day, and the orientation of aluminium capsules on the upper surface of the absorber would rather more effective than on the lower surface. In some other works, the effect of physical parameters of air and the orientation of fins on the performance of solar collector was also studied (El-Sebaii et al., 2011; Gulcimen et al., 2016). An experimental and theoretical investigation of a newly developed solar air collector was proposed (El-Sebaii et al., 2011; Gulcimen et al., 2016). Gulcimen et al., suggested different mass flow rates (0.012 kg/s, 0.026 kg/s, 0.033 kg/s) and orientation of fin (*α* = 30°, 45° and 60°) for obtaining the optimal efficiency of the collector. The result showed that the mass flow rate of 0.033 kg/s could maximize the solar collector efficiency up to 47%. Yang et al. (2012) examined the thermal performance of a single-pass collector using five different parameters. The parameters were heat transfer resistance, the height of the stagnant air film, properties of the clear glass, the emittance of the absorber plate and the thermal resistance of the backplate. The results showed that heat transfer resistance, the height of the stagnant air film and properties of the clear glass were the most significant parameters to improve the efficiency of the collector. However, an increase in the conductive thermal resistance of the backplate or decreasing the emittance of the absorber material had an insignificant effect on the energy efficiency.

The physical change in the solar collector merely provides the quantitative effect on the performance of a solar collector, which can be further analysed from a different perspective, such as the interaction of the control volume with the surrounding. In other words, the structural rearrangement of the solar collector is tested on an invariant flow property of medium (air) that might vary from one geographical location to another. For instance, the addition of fins might not be effective if the flow properties of the air change with time or local temperature. Apart from the temperature distribution within the collector system, the initial psychrometric of air also has a significant effect on the overall performance of a system. The increasing partial pressure of water vapour in the moist air can remarkably reduce the wet bulb depression (WBD). With this change, the quality of air available for the post-harvesting application also gets affected. However, the successive multi-pass collector system can condition the circulating air, but it also requires a higher storage temperature that can be achieved by using phase-change material. To determine the qualitative aspect of the thermal system, the exergy analysis is much more appropriate than a physical change in the configuration of the collector or increasing the mass density of the air. Various studies have been carried out on the exergy analysis of the solar air collectors besides energy analysis (Gupta and Kaushik, 2008; Hematian and Bakhtiari, 2015; Ho et al., 2005a). Gupta and Kaushik (2008) used the following parameters: aspect ratio, the mass flow rate per collector area and duct depth, to determine their effect on the exergy output rate. Their exergy analysis was merely based on the extensive properties of the medium used for the heating purpose. They noticed that the exergy could be augmented if the mass flow rate and the high aspect ratio. Their exergy analysis was pivoted on the extensive properties of the medium used for the heating purpose and thermodynamic properties of the medium was not involved. The different form of solar air heaters, single glass cover with fins, double glass without fins and double glass cover with fins, were investigated experimentally by Alta et al. (2010). The collectors tilted at angles 0°, 15° and 30° were examined at the mass flow rates of 25, 50 and 100 kg-s^−1^. It was concluded that the energy efficiency improves with the increased airflow rates, while the exergy efficiency decreased (Alta et al., 2010). They had concluded that the collector efficiency, the temperature difference of the air and pressure loss are the most critical parameters that impact the exergy loss in the collector.

The influence of global solar radiation intensity and the air mass flow rate on the collector absorber with a packed material was experimentally studied using the equations of the first and the second laws of thermodynamics (Bouadila et al., 2014). The daily average energy and exergy efficiencies were found to be 40% and 22%, respectively. Moreover, the outlet temperature of the collector was also influenced by the airflow rate (Bouadila et al., 2014). Dhaundiyal and Atsu (2020) determined the hydrodynamic effect of air on the performance of the system, and it was found that the orientation of the air influenced the thermal field formed on the surface of the energy system; however, psychrometric behaviour was overlooked during modelling the momentum and energy system with ODE45. The effect of flow type on the solar collector was studied, and it was observed that the active and passive flow both would contribute equally to enhance thermal efficiency growth rate (TEGR). It was noticed that active swirling of air surged TEGR by 23.83%, whilst it was 16.03% for passive swirling (Hu et al., 2020). However, the intrinsic factors related to air were not studied, and the focus was merely on the fluid mechanics of the circulating air. Likewise, in another study, the thermal behaviour of the solar collector was investigated from the quantitative aspect of airflow, and it was estimated that the relative increase of airflow from 0.006 to 0.02 kg-s^−1^ would increase the thermal efficiency of the solar collector by 43.36% (Rani and Tripathy, 2020). It was another quantitative approach to explain the thermal efficiency of the solar collector, but the essence of the discussion was devoid of the effect of air quality on the end use. From the literature review, it was concluded that the quantitative evaluation of the solar collector has been done and the study must be conducted from the perspective of the characteristics of the medium used for the desired purpose. The study focuses on the component-based analysis of the solar collector. Since the collector has been using for drying purpose, therefore involvement of psychrometric analysis becomes inevitable. The previous work is based on quantitative aspect of the collector, and none of them had focussed on the psychrometric behaviour of medium. The purview of the study is based on the thermodynamic study of the solar collector and it also considers the psychrometric characteristic of air. The proposed analysis is intrinsic in nature and it has not been covered in the previous work. 

In this study, the qualitative aspect of a solar collector was discussed for post-harvesting purpose. The objective of this work is to determine the exegetic aspect of a solar collector system having included the psychrometry of processed carrier fluid (air) that has been overlooked in the previous study (Prasad, 2013; Karwa and Chitoshiya, 2013; Ramadan et al., 2007; Dović and Andrassy, 2012; Ho et al., 2005a; Ho et al., 2005b; Omojaro and Aldabbagh, 2010; Krishnananth and Kalidasa, 2013; Gulcimen et al., 2016; Yang et al., 2012; Rani and Tripathy, 2020; Dhaundiyal and Atsu, 2020; Hu et al., 2020). To analyse, the system, the collector plate and the carrier fluid are considered as different control volumes. The exergy analysis of the carrier fluid (air) along with the effectiveness of the collector plate is examined for the steady-state, steady flow (SSSF) state of the air.

## Materials and methods

### The experimental unit under a pilot scheme

**Fig. 1:**
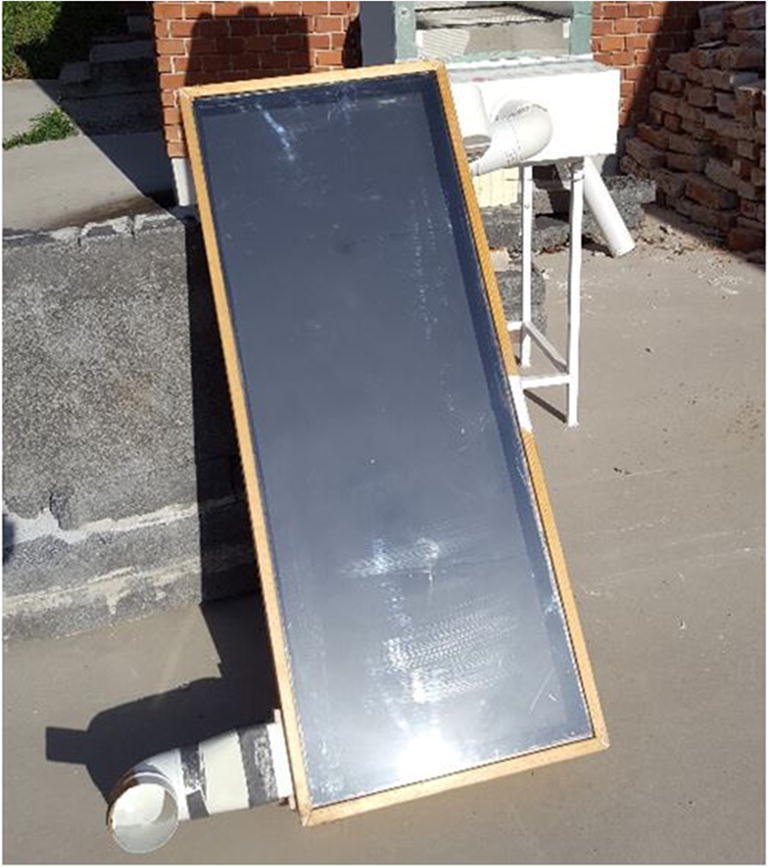
A flat plate solar collector retrofitted with a solar dryer

 A flat plate collector was critically examined in this analysis work. To prevent the heat loss from the front side to the ambient and also providing the sealing, a cell casting acrylic glass was used (it is a transparent diathermanous glass sheet, which allows the short wavelength radiation from the sun to the plate and is incapable of transmitting the low-temperature long-wavelength back to the ambience. Thus, the solar radiation trapped in a collector. Also, the heat loss from the carrier fluid to the ambient from the backside was prevented by using a layer of polystyrene. As a flat plate collector, a material with high absorptivity and low emissivity, copper, was used (Fig. 1). The flat plate solar collector used for experimental purpose is shown in Fig. 1. One of the surfaces of the copper plate was selectively coated with black enamel paint, whereas the other side was made reflective via polishing its surface. The passage of air duct was provided underneath the absorber plate. The rack angle of the solar collector was kept at 45° facing true south. The physical dimension of the proposed design was provided in Table 1. The regime of flow was examined by calculating Grashof and Reynold’s numbers for the carrier fluid (air) and the enclosed air space.

**Table 1:** Dimensions of the solar collector used for the analysis

Components	Length (L) (mm)	Width (W) (mm)	Height/ thickness (H) (mm)
Wooden frame	1200	500	150
Glass	1160	460	4
Copper plate	1160	460	1.2
Polystyrene	1160	460	80

**Fig. 2:**
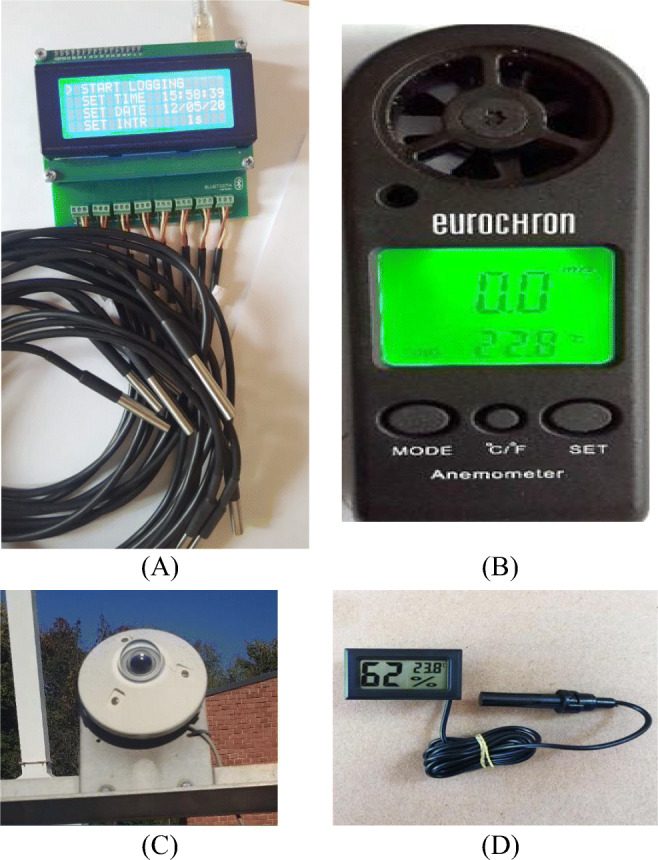
The equipment used for the thermal analysis (A — datalogger (4CH), B — handheld anemometer, C — pyranometer, D — digital humidity meter)

The geographical location of the setup is 47.4°N and 19.3°E. The experiments were conducted at the solar facility of the Szent Istvan University, Godollo, Hungary. The psychrometric as well as the thermal measurements were done under the no-load condition on the 25th of September 2019. The thermocouple ‘K’ (nickel-chromium) was used to measure the temperature at the inlet and the outlet of the collector. The temperature of the collector at the inlet of the air duct was considered to be equal to the ambient temperature. The duration of the measurement was from 10 A.M to 3 P.M. and based on 5-h testing, the exergy analysis was performed. The temperature sensors (DS18B20) with an accuracy of ±0.5°C from −10 to +85°C were used to store data using an 8-channel data logger. The global solar radiation was measured by a pyranometer with an accuracy of ±0.1Wm^−2^. For accessing the collected data, the solar radiation sensors were connected to the ADAMS 4018 interface that converts physical properties into digital signals in the data acquisition system. The velocity of a carrier fluid was computed by a digital handheld anemometer (Eurochron EC-MR 330) with ±0.3% accuracy. The psychrometric measurement of the ambient air was carried out with the help of a hygrometer. The detailed information about the instruments is provided in Table 2. The equipment used for the measurement of thermal properties is shown in Fig. 2.

**Table 2:** Equipment used for the data measurement

Instruments	Company, country	Specification (Dhaundiyal et al., 2021)
Handheld anemometer	EC-MR 330, Eurochron GmbH, Seebach, Germany	0 to 30 m-s^−1^
Pyranometer	CM-11, Kipp & Zonen, Italy	Max: 4000 W-m^−2^
Digital hygrometer	Diyomore, Hong Kong	10–99%
Data logger for temperature	4CH Temperature data logger, KRIDA Electronics, Latvia	−55 to 125°C
Data logger for global radiation	ADAM 4018, Advantech.co, Taipei, Taiwan	±6 µV/°C

### Thermal system analysis

**Fig. 3:**
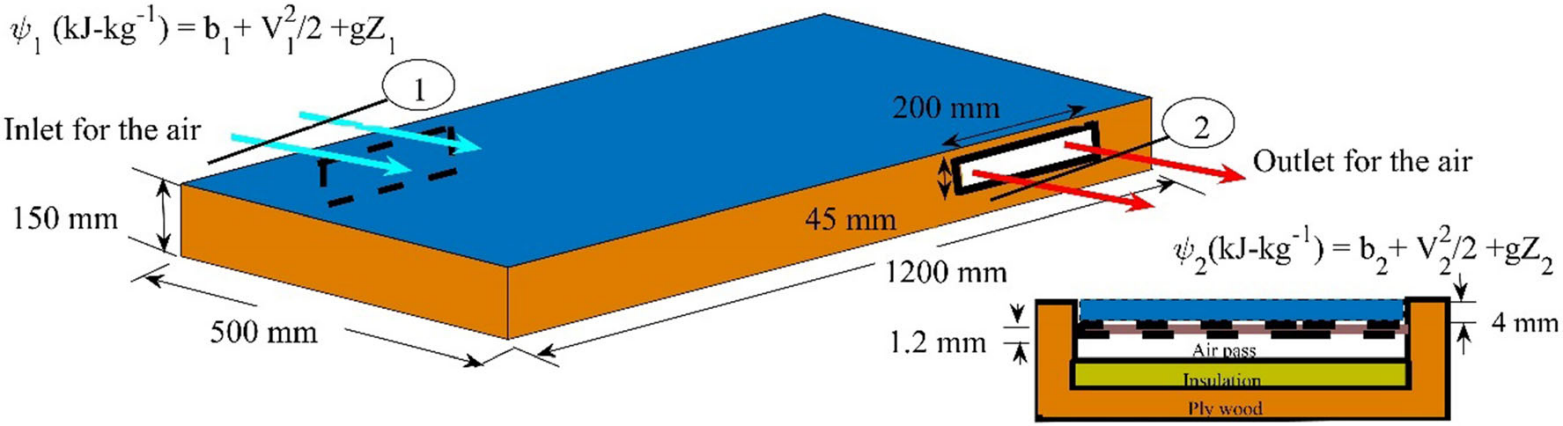
The geometrical dimension of a flat-plate solar collector

**Fig. 4:**
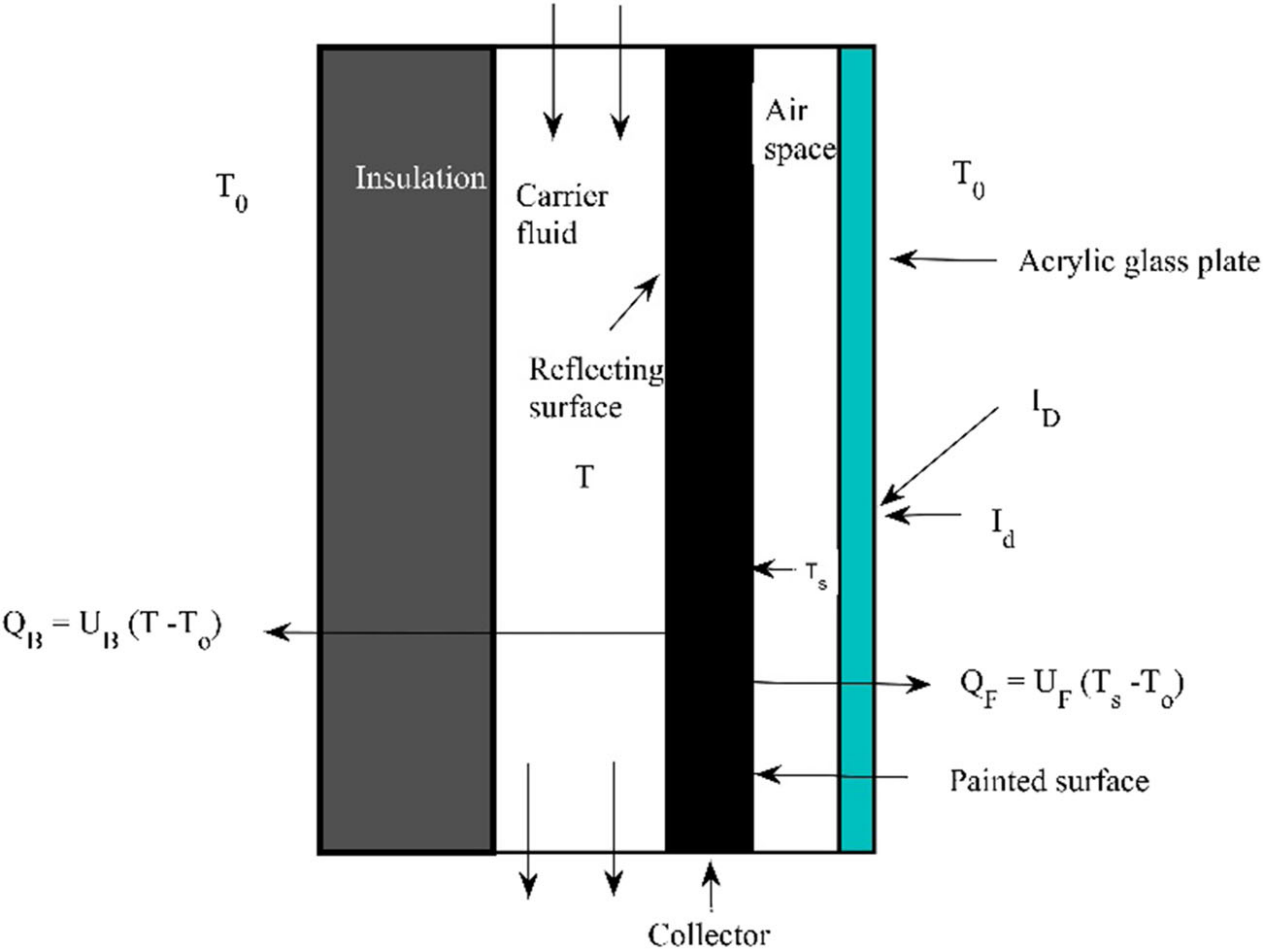
Schematic diagram of a flat plate solar collector

The detailed analysis imbibes the thermodynamic and heat transfer aspects of a solar collector system. The thermal performance of a solar collector facing the true south is investigated based on the law of entropy. The mass flow across the solar collector area is assumed to be constant, so the fluid is considered to be incompressible, steady, with no change in its hydrodynamic properties with temperature, and there is no increase in energy of the system $\frac{\partial E}{\partial \tau }=0$. The air is used as a carrier fluid, and the change in enthalpy is calculated by using the steady flow energy equation (SFEE). The exergy function is calculated at the inlet and outlet of the solar collector. The inlet and outlet ports are assumed to be the same in the cross-section, so the loss due to the pipe bending, sudden enlargement and sudden contraction are absent in the calculation. The rate of change in entropy of the collector plate is assumed to be zero $\frac{\partial s}{\partial \tau }$= 0. Table 3 shows the optical properties of the material used for the fabrication of the solar collector. The hydrodynamic/thermal properties considered for the analysis purpose are provided in Table 4. The overall heat coefficients at the front and back sides of the collectors determined to compute the change in the carrier fluid temperature over the surface area (*dA*) of the collector plate. The maximum temperature a collector plate can have is restricted by the stagnation temperature. The specific humidity (*ω*) of moist air is evaluated with the help of a psychrometric chart at DBT of 299.4 K. The ratio of the extended area to the solar collector area (*K*) is assumed to be unity. The coefficient for the sky radiation, *C*_s_(Stephenson, 1967), considered for the analysis is estimated through the interpolation for a particular day. The reveal height for the solar collection is 20 mm so that the solar light area (*A*_sun_) for a collector can be determined. The Gashof’s number for an inclined plate is calculated by multiplying the Gashof’s number for a vertical plate by sin (ϕ) of the tilt angle. The geometric dimension of the solar collector is shown in Fig. 3. The schematic diagram of the flat plate solar collector is shown in Fig. 4. The distance between the glazing sheet and the collector plate is 10.8 mm, and the area of the duct is 0.009 m^2^. Since fins are used, so the extended area is considered to be equal to the actual area of the collector (*K* =1). The time 0 s denotes 10 A.M, while 1800 s represents 3 P.M. The temperature measurement is done on an average basis.

**Table 3:** The optical and heat transfer characteristics of the materials used for manufacturing

Parameters	Glass (Dhaundiyal et al., 2021)	Copperplate (ASM International, 2002)	Polystyrene (Pelsmakers, 2019)	Plywood (The Building Regulations and Approved Documents, 2017)
*τ*_D_	0.90	-	-	-
*τ*_d_	0.94			-
*α*	0.05	0.62	-	-
*k* (W-m^−1^-K^−1^)	0.19	396.5	0.035	0.13

**Table 4:** The hydrodynamic/thermal properties of the air used for calculation (Arora, 1981)

C_p_	ν	ρ	ω_1_	*k*_a_
1.005 kJ-kg^−1^-K^−1^	16.2E-06 m^2^-s^−1^	1.17 kg-m^−3^	10.1 kg/kg d.a	0.027 W-m^−1^-K^−1^

The change in the exergy for the carrier fluid is given by Eq. (1). The *Q*_c_ is the heat transfer to the system at the heat storage temperature of *T*_r_

1 $$ \left({\psi}_1-{\psi}_2\right)=\left({b}_1-{b}_2\right)+\frac{\dot{Q_c}}{\dot{m}}\left(1-\frac{T_0}{T_r}\right) $$

The rate of entropy generation due to the collector plate is given by Eq. (2)

2 $$ \frac{\partial S}{\partial \tau }=\dot{S_c}+\sum \dot{\frac{Q}{T}} $$

The second law efficiency for the thermal system is given by Eq. (3)

3 $$ {\eta}_{II}={\eta}_c\left[\frac{\left(1-\frac{T_o}{T_s}\right)}{\left(1-\frac{T_0}{T_r}\right)}\right] $$

The following correlation is used for the enclosed air ($\overline{ha}$) (Eq. 4), whereas for the carrier fluid ($\overline{hf}$), Eq. (5) is considered for analysis purpose (Holman, 2002)

4 $$ \overline{h_a}=\frac{k}{L_t}\left[0.18\left(G{r}_a^{\frac{1}{4}}\right){\left(\frac{H}{L_t}\right)}^{-\frac{1}{9}}\right] $$

Equation (4) is valid for 2000 < *Gr*_*a*_ < 2 *x*10^4^

5 $$ \overline{h_f}=\frac{k}{L_e}\left[0.68+\frac{0.67{\left( Gr.\mathit{\Pr}\right)}^{\frac{1}{4}}}{{\left[1+{\left(\frac{0.492}{\Pr}\right)}^{\frac{9}{16}}\right]}^{\frac{4}{9}}}\right] $$

The validity of Eq. 5 is for *Gr*. *Pr*  < 10^9^

The heat $\dot{Q_c\ }$received by the collector plate is transferred to the carrier fluid and the ambient from the front side. After applying the energy balance, we get

6 $$ \dot{\dot{Q_c}=\left({A}_{sun}{I}_D{\tau}_D+A{I}_d{\tau}_d\right){\alpha}_s={U}_o{A}_o\left({T}_s-T\right)+{U}_FA\left({T}_s-{T}_o\right)} $$

Note: The detailed calculation is provided in the supplementary file

Note: Here, *Gr*_a_ is determined based on the thickness of the air space (*L*_*t*_), and *H* denotes the width of the solar collector. The detailed formulation for psychrometry and heat transfer analysis is provided in the supplementary file.

## Results and discussion

### Thermal characteristics of the solar collector

The detailed analysis of the solar collector system was performed based on the interaction of a thermal system with a carrier fluid (air) and its surrounding. The two different control volumes/surfaces were considered, and each has been examined separately. The heat transport across the thermal system was demarcated by the surfaces of the flat plate collector. The front and the back were surrounded by the enclosed air and a carrier fluid, respectively.

**Fig. 5:**
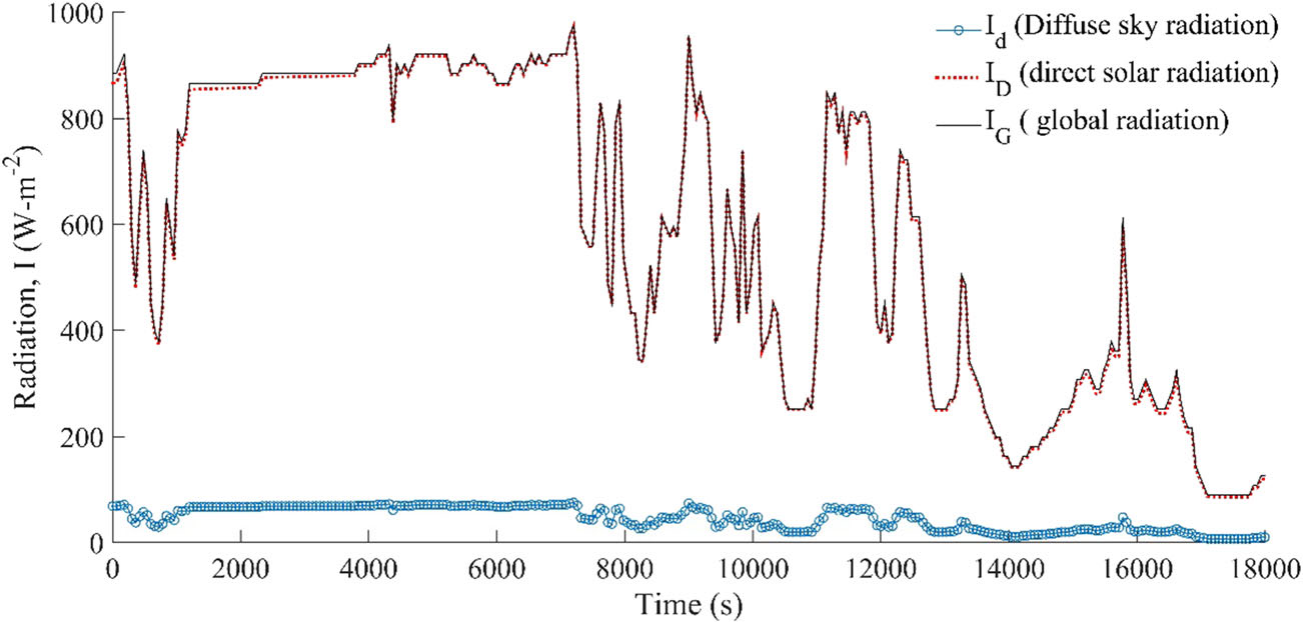
Variation in the radiation components with time

The variation in *I*_D_ (W-m^−2^) and *I*_d_ (W-m^−2^) within the selective duration (10 A.M to 3 P.M) is shown in Fig. 5. The quantitative change in the diffuse sky radiation as compared to the direct solar radiation was found to be nearly constant before noontime, and the fluctuation was commenced slightly before noon time (8000 s). On the other hand, the direct solar radiation changed constantly with time, and it decreased as the time proceeds after 1:17 A.M (10000 s). The overall variation in the direct solar radiation was noticed to be relatively high as compared to the diffuse sky radiation; however, the contribution of the diffuse sky radiation in estimating the collector efficiency would be very marginal, and the effectiveness of the solar collector mainly relies on the direct solar radiation and the geometric factor of the surface. The reason for undulation in the direct solar radiation (*I*_D_) is the dependence on the solar angles. Unlike the diffuse sky radiation which mainly depends on the angle factor (*F*_ss_) between the surface and the sky and the sky radiation coefficient, the direct solar radiation is altered with the direction of facing and the altitude angle of a surface from the sun. The direct radiation would be maximum when either declination angle would be minimum (or |*β* − *φ*| is minimum) or the altitude angle of a surface is maximum (or deviation between |*l* − *d*| tends to be minimum). On the other hand, the diffuse sky radiation is constant for a particular day, and it relies on the angle factor, which mainly varies with the rake angle of the surface. Thus, a single factor, which is constant for a typical solar collector, hardly impacts the quantitative measurement of the diffusion sky radiation on the surface. The overall deviation between *I*_G_ and *I*_D_ is marginally very low, and the contribution of the diffusion sky radiation in the energy analysis also depends on the diffuse transmissivity of material used for sealing purpose, i.e., diathermanous film layer.

**Fig. 6:**
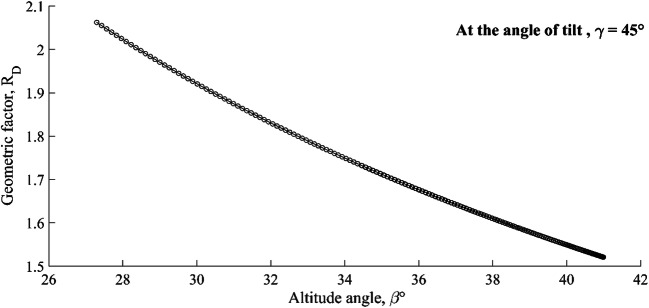
The change in the geometric factor (RD) for the collector surface to the altitude angle, β

**Fig. 7:**
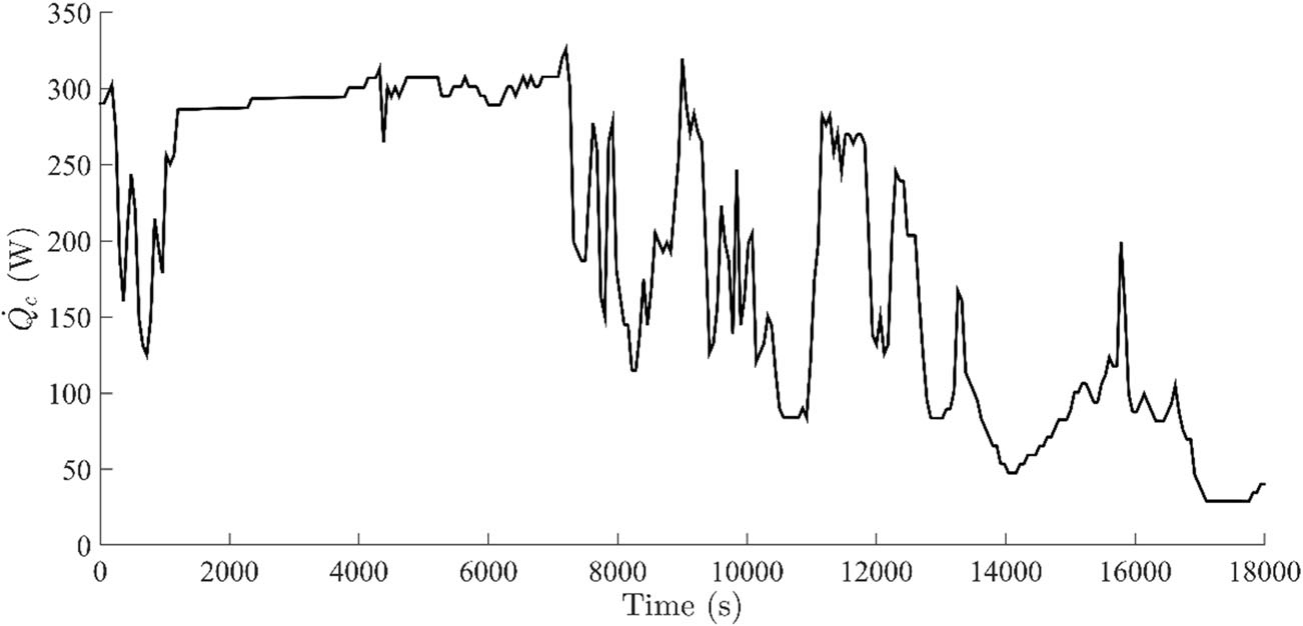
The rate of change of heat energy of a collector plate with time

Another governing factor that increases the relative competitiveness of a solar collector in terms of the tilt angle, the altitude angle, and the azimuth angle is the geometric factor (*R*_D_). The change in the geometric factor of the solar collector with *β* is illustrated in Fig. 6. A constantly decreasing magnitude of the direct solar radiation can also be related to the rake angle of a solar collector. Therefore, the effect of the geometric factor on the solar intensity was also included, and it was found that the geometric factor for the given pilot unit is decreasing constantly with increasing altitude angle at a constant tilt angle. The change in the geometric factor might also be one of the reasons for the inefficient utilisation of the incoming radiation. The optimisation of the geometric factor (*R*_D_) can also influence the exergy of a solar collector. A constant decrease in the geometric factor reduced the overall transmission of the incoming radiation across the acrylic layer. A geometric factor will provide its optimum advantage when the relative deviation between φ and *β*$\left|2\beta -\varphi \right|<\frac{\pi }{2}$ or tilt angle should be *γ* < |*π* − 2*β*| at noontime. Thus, it can be concluded that the relative variation of a tilt angle γ to the altitude *β* might reduce the perturbance in the solar intensity, as well as the rate of heat transfer by the collector plate, will be least impacted. The variation in the heat energy $\dot{\left({Q}_c\right)}$ transmitted by the collector plate is shown in Fig. 7. The fluctuation pattern in the heat transfer across the copper plate was similar to that seen in the case of direct solar radiation (*I*_D_). The only difference was the quantitative variation in the heat transport across the plate. Similarly, the gain of heat energy was seen to be maximum before noon, and it started dwindling after noon time (10000 s). Quantitatively, the solar intensity could be utilised to the utmost degree if the *A*_sun_ collector area receiving the direct radiation or the ratio *K* (*A*_0_/*A*) are increased to some extent. The extended area can be increased by using a corrugated plate (Dović and Andrassy, 2012) rather than a flat plate, or the fins are selectively provided to the surface which is exposed to the carrier fluid (but the surface should be reflective to increase the radiosity of the surface or reduce the space resistant to radiant energy). For the given thermal system, the transfer of heat energy across the plate was constantly decreasing with time, and the average loss of the solar collector was computed to be 0.37 kW per unit area of the solar collector.

**Fig. 8:**
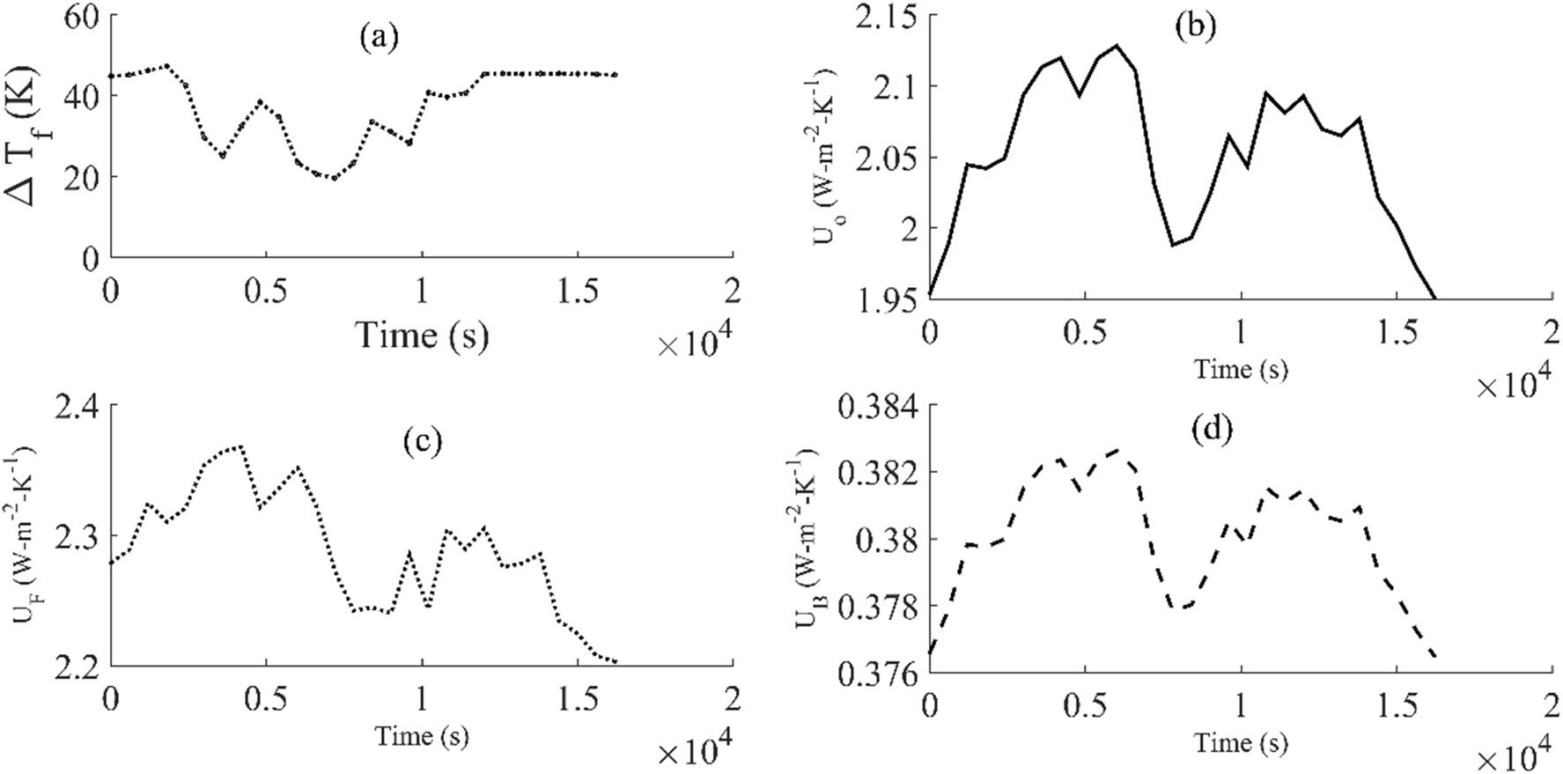
The change in the overall heat transfer coefficients (U) and temperature (Tf) of the carrier fluid with time (a: Tf, b: U0, c: UF, d: UB)

The factors that affect the heat transfer across the collector plate are provided in Fig. 8a–d. The relative change in the carrier fluid temperature (Fig. 8a) with the surface temperature of the collector plate *T*_s_ is 6–13%, whereas it is 7–16% with ambient temperature *T*_o_. The overall heat transfer coefficients from the front (*U*_F_) (Fig. 8c) and back (*U*_B_) (Fig. 8d) sides of a collector were found to be 2.28 W-m^−2^-K^−1^ and 0.38 W-m^−2^-K^−1^, respectively, whereas the overall heat transfer coefficient from the copper plate to the carrier fluid is 2.051 W-m^−2^-K^−1^. The relative deviations of the heat transfer coefficients *U*_F_ and *U*_B_ with respect to *U*_0_(Fig. 8b) are 9–16% and 80–82% respectively. The heat transfer to the ambient from the front side is 20–32% higher than that of the heat transfer to the carrier fluid for a unit increase in temperature of the carrier fluid and ambient air. The radiant energy transferred to the enclosed air was 38% (146 W-m^−2^) of the total incoming radiation transmitted through the acrylic glass (plexiglas G), whereas 65% (95 W-m^−2^) was transferred back to the ambient through the glass and the rest (51 W-m^−2^) was trapped in the air space. Apart from the emission of solar radiation from the top surface of the copper plate, the average heat transfer coefficient of the enclosed air $\overline{h_a}$ was also 43–52% higher than that of the carrier fluid ($\overline{h_f}$). Thus, the rate of dissipation of heat was also found to be different on both sides of the collector plate. The reason for the decrease in the convective resistance to the heat flow is the thickness of the trapped air for the constant value of thermal conductivity. It will have more impact on the convective heat transfer than a change in the Grashof number. It also implies that heat transfer at the interface of the collector plate and the trapped air is dominated over the heat transfer in bulk. The overall heat transfer coefficient *U*_0_ at the beginning of operation of the solar collector is relatively very low (Fig. 8b), and it increases steeply within a duration of 5000 s and remains steady for 1000 s. Though the *U*_0_ was lower than *U*_F_ in magnitude, the standard deviation in the values of *U*_0_ was 12.5% higher than *U*_F_, which implies the change in the rate of heat energy collection to the carrier fluid was relatively higher than that to the air space. Similarly, the overall heat transfer coefficient (U_B_) for the solar collector has a similar variation pattern with *U*_0_, but the relative deviation is marginally low, i.e., 0.0018 W-m^−2^-K^−1^. It shows that the heat transport corresponding to a unit increase in the temperature of the carrier fluid is appreciably low for polystyrene.

**Fig. 9:**
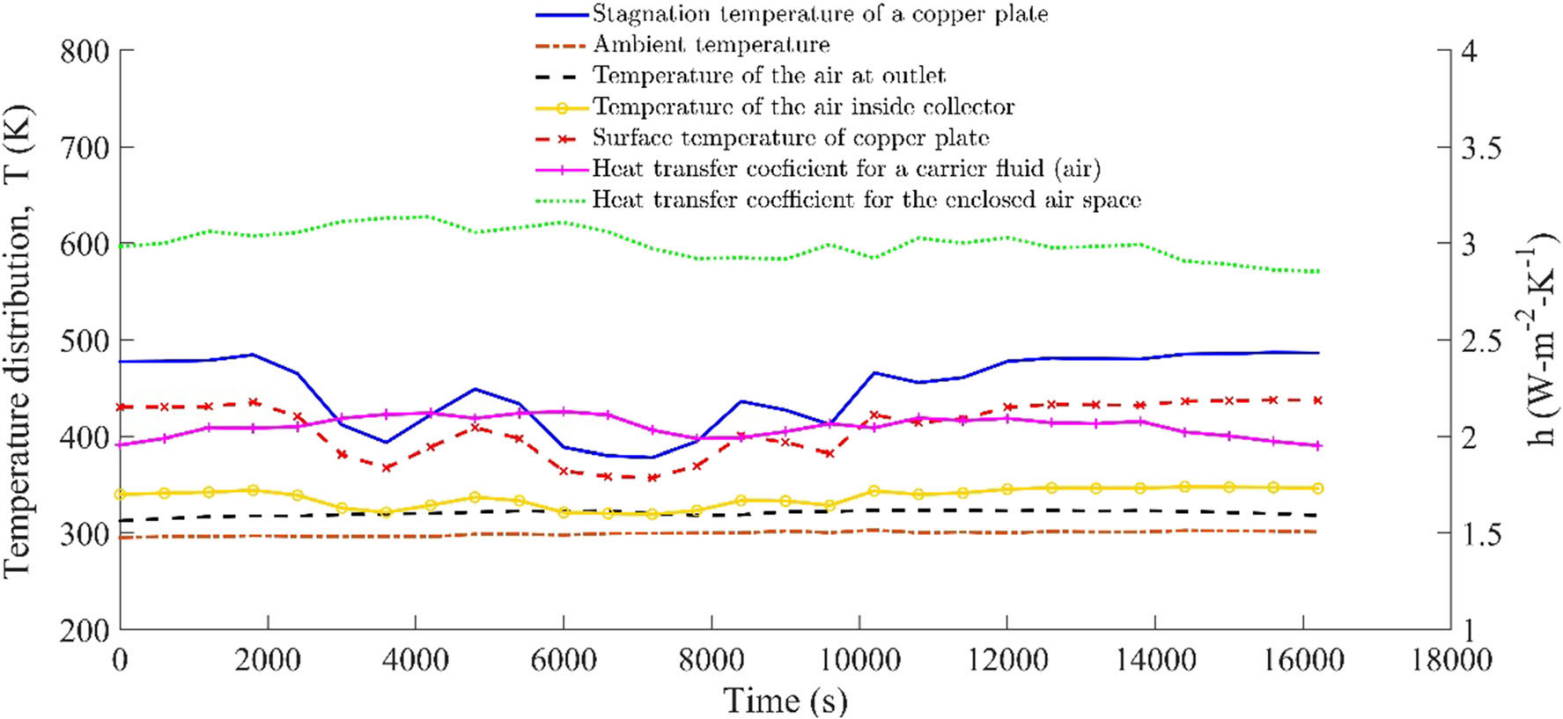
The temperature of air and the copper plate with the heat transfer coefficient of enclosed/ flowing air

The temperature distribution and the heat transfer coefficients of the air space (*h*_a_) and the carrier fluid (*h*_f_) are illustrated in Fig. 9. The ambient temperature at the outlet of the carrier fluid duct was nearly constant throughout the heating process. Similarly, the variation in the ambient air temperature shown the same thermal characteristics but differ quantitatively from the actual temperature of the collector plate, the outlet duct, and the stagnant temperature of the copper plate surface. The relative deviation of the temperature of the carrier fluid at the outlet of the solar collector with ambient temperature was found to vary from 5.7 to 8.22%, whereas it was 9.8–27% to the surface temperature of a collector plate and −0.9 to 8.1% to the heated carrier fluid temperature *T*_f_. A temperature drops in the carrier fluid, and the collector plate was seen during the noontime (12.06 P.M to 12.22 P.M). This happened due to a sudden decrease in the overall heat transfer coefficients (*U*_F_, *U*_B_ and *U*_o_) or an increase in the convective heat transfer resistance of the carrier fluid. A sharp reduction in the heat transfer coefficients can also be noticed in Fig. 8. The stagnant temperature for the given solar collector was also found. It is a maximum temperature of a collector plate that can be attained by the flat plate collector, which was 6–11% higher than the actual temperature a solar collector could have for a stagnant mass ($\dot{m}=0\Big)$ or $\left(\dot{Q_f}=0\right)$of the carrier fluid. This can be attained for a carrier fluid flowing over the collector plate if the rate of change of the momentum of the carrier fluid is equal to the net external pressure force, or for a steady flow of a carrier fluid in the absence of the external pressure force, and it would be independent of the extended area used. An asymptotic approach can also be adopted if the thermal boundary layer thickness *δ*_*th*_ → 0 or thermal diffusivity of a plate to be very small for a constant hydrodynamic boundary layer, *δ*, i.e., near the laminar sub-layer of the carrier fluid (the velocity gradient remains constant over the characteristic length, *L*_e_, and the flow rate will be dominated by the shear stress of the surface.

**Fig. 10:**
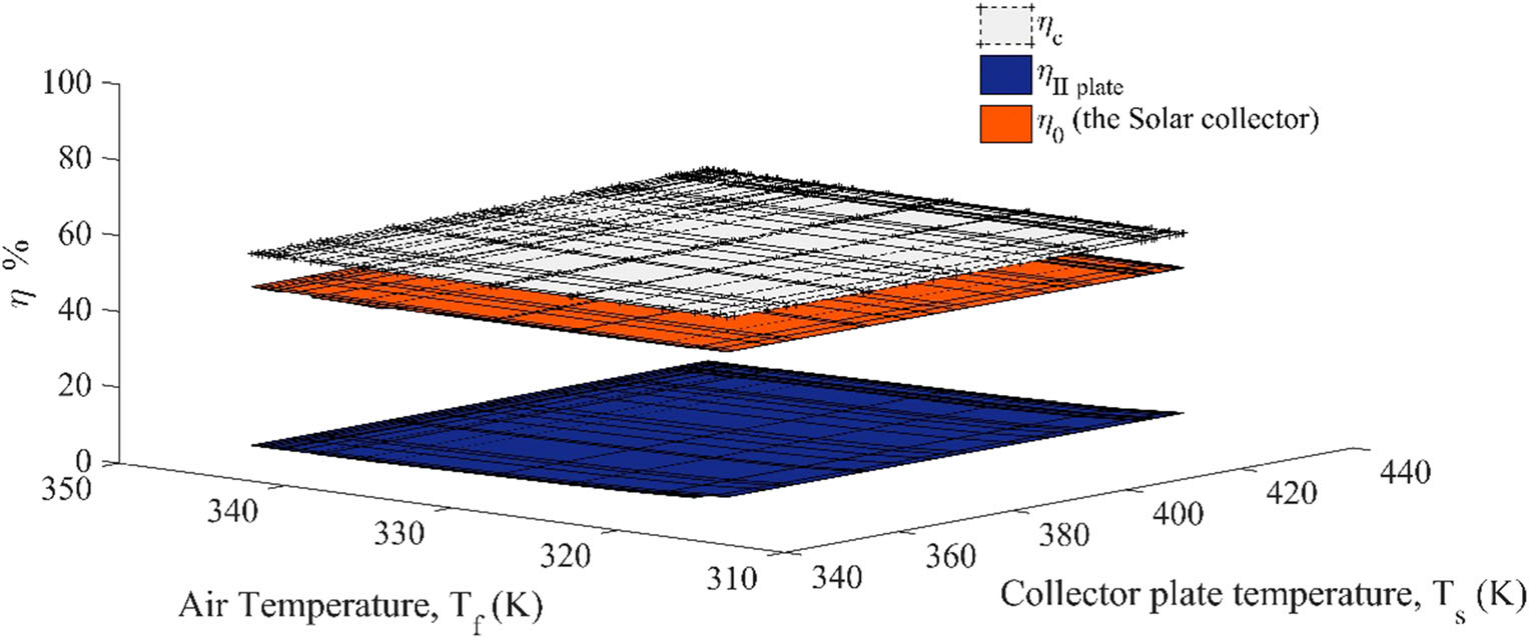
Variation of collector efficiency (η, ηII) with the carrier fluid (air) and the collector plate temperature

The effect of the temperature distribution across the carrier fluid and the collector plate was examined by demarcating the boundaries of the system. The variation in the copper plate efficiency (*η*_c_%), the second law efficiency and the solar collector for the given thermal system with the temperature of the carrier fluid temperature (*T*_f_) and its surface temperature are illustrated in Fig. 10. The average collector efficiency (*η*_0_) was found to be 41%, whereas the second law efficiency (*η*_II_%) for a given design was in the range of 22–25%. The first law efficiency (*η*_c_%) of the copper plate varied in the domain of 51–52%, which was relatively 24 to 26% higher than the overall efficiency of the solar collector. On the contrary, the exergetic efficiency of the heating material (*η*_II_%) (the Cu plate) was 28.29–33% (Table 5) for heating the carrier fluid. The exergy loss by the collector plate was partially lost to the ambient from the front and the rear sides of the solar collector, whereas the rest was used to increase the temperature of the carrier fluid and the enclosed air space. The variation pattern was similar for the solar collector and the first law efficiencies of the collector plate. A similar deviation was seen during the decrease in the surface temperature of the collector plate (*T*_s_) and the temperature of the carrier fluid (*T*_f_). A significant change in the solar collector efficiency η_0_% was noticed during noontime, which varied in the range of 0.4–1.4%, whereas the drop in the collector plate efficiency *η*_c_% during the same time was 0.005–0.007%. The collector plate and carrier fluid were considered the two control volumes, which interact differently with their surroundings. Based on the same concept, the overall exergy analysis of the solar collector was performed.

**Table 5:** Thermodynamic parameters for the collector plate

Parameters	Collector plate	Range of variation
***η***_***c***_	51.7%	51–52%
***η***_***II***_	31.20%	28.29–33%
$\dot{{\boldsymbol{Q}}_{\boldsymbol{c}}}$	238.32 W	28.6–326 W
***I***_***D***_	582.63 W-m^−2^	85.65–973 W-m^−2^
***I***_***d***_	45.41 W-m^−2^	6.97–75.31 W-m^−2^
***T***_***st***_	448.46 K	378–487 K
***T***_***s***_	408.84 K	357–438 K
*T*_*r*_	537.32 K	425–600K
***R***_***D***_	1.64	1.52–2.06
*η*_*0*_	41%	40–42%
$\dot{{\boldsymbol{S}}_{\boldsymbol{c}}}$	0.13 W-K^−1^	0.04–0.18 W-K^−1^
*A*_*Sun*_	0.59 m^2^	-

**Fig. 11:**
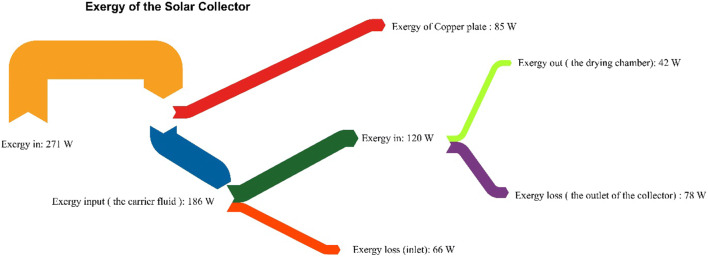
Grassmann diagram (exergy) for the flat plate collector

The Grassmann diagram for the solar collector is illustrated in Fig. 11, where an exergy of 271W denotes the total average exergy derived through the global radiation. Similarly, the exergy of the copper plate indicates that the actual exergy was utilised by the copper plate to increase its heat capacity, while the rest 186 W was provided to increase the exergy of the carrier fluid and the ambient. Out of the 186W, the carrier fluid consumed 67% of exergy for increasing its heat capacity and transferring the heat energy to the ambient via the backside of the solar collector. The carrier fluid also gained some energy due to datum and dynamic pressure; however, it was relatively very low as compared to the external heat transfer by the collector plate. The net exergy available for the end application was 15% of the total exergy provided to the solar collector, 23% of the exergy provided by the collector plate and 35% of the total exergy gain by a carrier fluid during the heating process. The exergy loss during the heating process was highest at the interface of copper plate and carrier fluid, followed by the exergy losses at the outlet and the inlet of a collector. It can be concluded that the copper plate can be rather best utilised for the double pass than a single pass thermal system where the carrier fluid is allowed to flow onto both sides of the collector plate in a counter-clockwise direction. The losses at the inlet and outlet can be prevented by using proper thermal insulation so that the mixing of the air streams does not happen. The rate of entropy generation $\dot{S_c}$ due to the heat transfer through the copper plate was found to be 0.13 W-K^−1^, whereas the entropy generation $\dot{S_f}$ was 0.17 W-K^−1^ for the carrier fluid (Table 6). The Keenan function at the outlet of the collector was estimated to be −0.54 kJ-kg^−1^. An exergy loss has happened due to the volumetric expansion of the carrier fluid at the outlet of the collector, which eventually decreased the enthalpy and entropy of the system. The change in the net entropy of the solar collector was calculated to be 0.069 kJ-kg^−1^-K^−1^. The irreversibility of the system was higher in the carrier fluid than that for a collector plate. The net exergy output of the carrier fluid was 34% of the total exergy provided during the processing time. Detailed information about the solar collector plate and the carrier fluid is provided in Tables 5 and 6.

**Table 6:** Thermodynamic/heat transfer parameters for the carrier fluid

Parameters	Carrier fluid	Range of variation
***η***_***IIf***_	34%	31–35%
***ΔH***_**1**_	37.72 kJ-kg^−1^	19.77–47.53 kJ-kg^−1^
***−ΔH***_**2**_	16.44 kJ-kg^−1^	3–28.06 kJ-kg^−1^
***b***_**1**_	2.28 kJ-kg^−1^	0.62–3.42 kJ-kg^−1^
***−b***_**2**_	0.54 kJ-kg^−1^	6.1E-04–1.16 kJ-kg^−1^
*ψ*_1_	23.12 kJ-kg^−1^	7.82–33 kJ-kg^−1^
*ψ*_2_	7.86 kJ-kg^−1^	7.28–8.44 kJ-kg^−1^
***H***_***fw***_	331.8 kJ-kg^−1^	328–336 kJ-kg^−1^
***H***_***fa***_	427.63 kJ-kg^−1^	419.14–431.11 kJ-kg^−1^
Δ*S*_f_	0.069 kJ-Kg^−1^-K^−1^	0.06–0.08 kJ-Kg^-1^-K^−1^
***ω***_***fw***_	10.1 g/kg d.a	-
***ω***_***fd***_	34 g/kg d.a	-
$\dot{{\boldsymbol{S}}_{\boldsymbol{f}}}$	0.17 W-K^−1^	0.01–0.42 W-K^−1^
*ϕ*_wf_	42%	34–64%
*ϕ*_*df*_	4.8%	4.6–5.1%
*T*_f_	337 K	319.37–348 K
***U***_**0**_	2.051 W-m^−2^-K^−1^	1.95–2.13 W-m^−2^-K^−1^
***U***_***F***_	2.28 W-m^−2^-K^−1^	2.2–2.36 W-m^−2^-K^−1^
***U***_***B***_	0.38 W-m^−2^-K^−1^	0.37–0.383 W-m^−2^-K^−1^
***Gr***_***f***_	1.67E+08	1.36E+08–1.93E+08
***Gr***_***a***_	9560.55	7803–11382
***∗Gr***_***f***_***.Re***^***−*2**^	0.40	0.30–0.43
$\dot{{\boldsymbol{m}}_{\boldsymbol{f}}}$	5.21 g-s^−1^	2.2–7.6 g-s^−1^

^*^It happens when air flows over a heated surface at a low velocity

The solar collector was used for a drying process; therefore, a psychrometric analysis was also carried out, and it was found that the relative humidity of the processed carrier fluid (air) was reduced by 37.2%. On the other hand, the specific humidity or humidity ratio of the carrier fluid was increased by 24%. It implies that the specific volume of air for the same mass flow rate of the carrier fluid is increased at the outlet of the solar collector; therefore, the temperature drop was observed at the outlet duct of the collector. The comprehensive formulation for psychrometry is provided in Appendix A. One more thing is to be noted that the flow regime of the carrier fluid across the solar collector was found to be mixed (free and forced convection) flow. Therefore, the sudden drop and rise in the carrier fluid temperature were seen. The change in the flow regime happened since the velocity of the flow of the carrier fluid was considerably low (0.2–0.7 m-s^−1^). Hence, the energy losses across the various components of the solar collector were also not steady with time. Some comparisons were also made with different types of solar collectors (Table 7).

**Table 7:** Comparison of the different types of collector plates

Design	Fluid passage	Solar collector efficiency (*ηc*)
Flat plate Collector (pilot scheme)	Single pass	41–42%
Triangular cross-section(Hematian and Bakhtiari, 2015)	Double pass	55.4%
Wire mesh between the fins (El-khawajah et al., 2011)	Double pass	75%
Steel wire mesh (Omojaro and Aldabbagh, 2010)	Single pass	59.62%
Wire mesh (Aldabbagh et al., 2010)	Single pass	45.93%
With finned and V-corrugated (El-Sebaii et al., 2011)	Double pass	55.7 and 65.2%
Longitudnal semi-cylindrical fins (Chabane et al., 2014)	Single pass	40.02% with fins and 34.12% without fins

## Conclusion

The comprehensive analysis of a solar collector based on the system and surrounding interaction and its effect on the exergy of the sub-components. The carrier fluid and collector plate were separately examined, and their relative effectiveness was estimated with the help of the thermodynamic laws and heat transfer application. The range of variation in the solar collector efficiency over a selected duration is 41 to 42%, whereas the exergetic efficiency (*η*_II_) varied from 28.39–33%. The exergy of the carrier fluid obtained for the collector was 7.86 kJ-kg^−1^. The energy loss across the collector plate was 48–49% of the total radiant energy received by the solar collector. The enthalpy of the processed carrier fluid was in the domain of 419.14–431.11 kJ-kg^−1^. The rate of entropy generation $\backslash \mathrm{dot}\left\{\mathrm{S}\_\mathrm{f}\backslash \right\}$ estimated from the Gouy -Stodola’s law for the carrier fluid was 0.17 W-K^−1^. Similarly, the entropy generation $\dot{S_c}$ due to the heat transfer across the collector plate is found to be 0.13 W-K^−1^. The relative increase in the irreversibility of the system due to the carrier fluid was 30%. From Grassmann diagram, the exergy loss of a carrier fluid was estimated to be 65% to the ambient at the inlet of dryer, whereas it was 35% at inlet of the collector. Similarly, the collector plate was merely able to hold 31% of total exergy provided to the system, while the rest 68.63% was given in to carrier fluid and enclosed air. While flowing across the collector, the carrier fluid lost 35.48% to ambient via insulation. So, the net exergy available at the end use was solely 15.49% of the net exergy provided to the solar collector system via radiation. If the overall assessment of exergy is carried out, it could be seen the carrier fluid held the mammoth share of exergy that was lost during the flow. So, the lost exergy could be utilised to precondition the inlet air.

The maximum permissible temperature of the collector plate (*T*_st_) for a given $\dot{Q_c}$ varied from 378 to 487 K. The relative change in the relative humidity of the carrier fluid during the heating process was 89%. Apart from the energy analysis, the geometric factor provides its maximum contribution to the direct solar radiation if the deviation between altitude angle and angle of the surface from vertical is $\left|2\beta -\varphi \right|<\frac{\pi }{2}$ at the noon. The direct radiation can be maximum when |*β* − *φ*| is minimum or the altitude angle of a surface is maximum. In contrast, the diffuse sky radiation is fairly constant for a particular day, and it depends on the tilt angle of the surface. The overall heat transfer coefficient (*U*_F_) between the collector plate and the ambient had deviation of 9–16% with respect to *U*_0_.The stagnant temperature ((*T*_st_) for a given design was estimated to be 6–11% higher than the actual collector temperature (*T*_s_) in a steady state of the carrier fluid. The mass flow rate of the carrier fluid will be more likely to be maximised as the overall thermal residence between plate and ambient, and plate and fluid gets minimised. Some suggestion for the improvement of an existing system is based on the exergy analysis.

1. The air space thickness should be comparable to the characteristic length of a collector plate so that convective heat loss (1≤ Bi) is minimised from the front side of a collector.
2. The exergy of the flat collector can be utilised far more efficiently if the single counterflow passages are provided on each side of the absorber plate so that the thermal deviation would remain least between the reservoir temperature and the temperature at which the heat energy is absorbed by the carrier fluid.

## Supplementary Information

ESM 1 (DOCX 22 kb)

## Notations

Symbol: Description, Unit
*d*: Declination angle, degree
*β*: Altitude angle, degree
*α*: The azimuth angle of a collector, degree
*l*: Latitude of the location, degree
*k*: Hour angle, degree
*φ*: The tilt angle of the surface with the vertical, degree
*R*_D_: Geometric factor, -
*I*_D_: Direct solar radiation, W-m^−2^
*I*_d_: Diffuse sky radiation, W-m^−2^
*I*_n_: Global radiation, W-m^−2^
*F*_sg_: Angle factor, -
*C*_s_: The coefficient for the sky radiation, -
*Q*_c_: Heat received by a collector plate, W
*Q*_f_: Heat gain by a carrier fluid (air), kJ-kg^−1^
*H*: Enthalpy of a carrier fluid, kJ-kg^−1^
*b*: Keenan function, kJ-kg^−1^
*ψ*: Exergy function, kJ-kg^−1^
$\dot{{\boldsymbol{S}}_{\boldsymbol{w}}}$: Rate of entropy generation in the collector plate, W
Δ*S*_f_: The change in entropy of a carrier fluid, kJ-kg^−1^
$\dot{{\boldsymbol{S}}_{\boldsymbol{f}}}$: Rate of entropy generation due to loss of exergy of a carrier fluid, W
*T*_0_: The temperature of the ambient air, K
*T*_f_: A carrier fluid temperature, K
*T*_s_: The temperature of the collector plate, K
*T*_st_: The stagnant temperature of the collector plate, K
*T*_r_: The temperature of the reservoir, K
*U*_*0*_: The overall heat transfer coefficient from a collector plate to the carrier fluid, W-m^−2^-K^−1^
*U*_F_: The overall heat transfer coefficients from a collector plate to the ambient, W-m^−2^-K^−1^
*U*_*B*_: The overall heat transfer coefficients from the carrier fluid to the ambient, W-m^−2^-K^−1^
*I*_D_: The direct solar irradiance, W-m^−2^
*I*d: The diffuse solar irradiance, W-m^−2^
*h*: Convective heat transfer coefficient, W-m^−2^-K^−1^
*Gr*: Grashof’s number, -
*Re*: Reynold’s number, -
*Pr*: Prandtl number, -
*η*_*c*_: The first law efficiency of the collector plate, -
*η*_*II*_: The second law efficiency of the thermal system, -
*η*_0_: the solar collector efficiency, -
*ω*: Specific humidity, kg/kg d.a
*A*_sun_: Collector surface area exposed to I_D_, m^2^
*C,f*: The subscript for a collector plate and a carrier fluid respectively, -
*α*: Absorptivity, -
*τ*_D_: Transmissivity for the direct radiation, -
*τ*_d_: Transmissivity for the diffuse radiation, -
*k*_a_: Thermal conductivity of air, W-m^−1^-K^−1^
*A*_0_: Extended area, m^2^
*L*_*e*_: Characteristic length, m
*σ*: Thermal expansion coefficient, K^−1^
*ν*: Kinematic viscosity, m^2^-s^−1^
*V*: Velocity of air, m-s^−1^
*Z*: The vertical height of the surface of a collector, m
*ϕ*: Relative humidity of the air, %
*d,w*: Processed and unprocessed air respectively, -
*F, B*: Subscripts denote the overall heat transfer coefficients for the front and back sides of a plate respectively
