# Supplementary material for: The effect of psychrometry on the performance of a solar collector
Source: Environ Sci Pollut Res Int. 2021 Sep 30;29(9):13445–58. doi: 10.1007/s11356-021-16353-5 (PMC8803786; doi:10.1007/s11356-021-16353-5)
Supplement: Supplementary file 1 — (DOCX 22 kb) [file 11356_2021_16353_MOESM1_ESM.docx]

The Effect of Psychrometry on the Performance of a Solar Collector

^*^Alok Dhaundiyal^1^, Gedion G. Habtay^1^

^1^*Institute of Process Engineering, Szent Istvan University, Godollo, Hungary*

Corresponding author :*Dhaundiyal.Alok@phd.uni-szie.hu

**Appendix A**

After energy balancing on both the sides of the plate, we get Eq. (A.1)

$\dot{\dot{Q_{c}}=\left( A_{sun}I_{D}\tau_{D}+AI_{d}\tau_{d} \right)\alpha_{s}=U_{o}A_{o}\left( T_{s}-T \right)+U_{F}A(T_{s}-T_{o})}$ (A.1)

In Eq.(A.1), *U*_0_ represents the overall heat transfer from a collector plate to a carrier fluid, whereas *U*_F_ and *U*_B_ denote the overall heat transfer coefficients from plate to ambient (from the front side) and from carrier fluid to the ambient( from the back side)

Let us assume the temperature of fluid flowing over the elemental area d*A* is heated by d*t*, then

$\int_{T_{1}}^{T_{2}} \dot{m}CdT= \int_{0}^{A} \frac{U_{o}\left( T_{s}-T \right)}{K} dA-\int_{0}^{A} U_{B}\left( T-T_{0} \right)dA$ (A.2)

Substitute the value of $t_{s}$ from Eq.(A.1) to Eq.(A.2)

$T_{s}=\frac{\dot{\left[ \left( \frac{\dot{Q_{c}}}{A_{0}} \right)+\left( U_{o}T+KU_{F}T_{0} \right) \right]}}{U_{0}+KU_{F}}$ (A.3)

After eliminating T_s_ from Eq. (A.2), integrate the Eq.(A.4), we have,

$\int_{T_{1}}^{T_{2}} \frac{{-N}_{2}dT}{N_{1}-N_{2}\left( T-T_{0} \right)}= -N_{3}$ (A.4)

Here, $N_{1}= \frac{\left( \frac{\dot{Q_{c}}}{A} \right)}{\left[ 1+\left( \frac{U_{F}}{U_{o}} \right)K \right]},N_{2}= \frac{\left( \frac{\dot{Q_{c}}}{A} \right)}{\left[ 1+\left( \frac{U_{F}}{U_{o}} \right)K \right]}+U_{B}$,$N_{3}= \frac{AN_{2}}{\left[ \dot{m}C \right]}$

After integrating Eq.(A.4), we have

$\Delta T_{f}=\left( T_{2}-T_{1} \right)=\left[ \frac{N_{1}}{N_{2}}-\left( T_{1}-T_{0} \right) \right](1-e^{-N_{3}})$ (A.5)

In the given case, $T_{1}=T_{0}$, so the final temperature of a carrier fluid will become,

$T_{2}=T_{0}+\left[ \frac{N_{1}}{N_{2}}-\left( T_{1}-T_{0} \right) \right](1-e^{-N_{3}})$ (A.6)

For the maximum value of T_2_, $N_{3}\to\infty$ , so that $\dot{m}=0$. Under this situation, $\dot{Q}=0$ and $T_{2}=T_{1}=T_{s}$. For K =1, the expression will be

$\left( U_{F}+U_{B} \right)\left( T_{st}-T_{0} \right)= \frac{\dot{Q}}{A}$ (A.7)

Thus, we get the stagnation temperature of a collector plate.

Similarly, psychrometric analysis of the thermal system is done by balancing moisture and the dry air

Applying continuity equation at inlet and outlet, we get

$$\dot{m}_{a_{1}}= \dot{m_{a_{2}}}=\dot{m_{a}}$$

The mass of water gets condensed during the temperature drop at the outlet pipe.

$$\dot{m_{w}}=(\omega_{fw}-\omega_{fd})\dot{m_{a}}$$

For knowing the specific humidity or humid ratio of the dried air, the steady flow energy equation for the dehumidification process is applied for 1 kg of dry air.

$\dot{Q_{c}}+\dot{m_{a}} H_{1}=\dot{m_{a}}H_{2}+\dot{m_{w}}H_{w_{2}}$ (A.8)

Here, $\dot{m_{w}}$is the mass of the condensed water vapour.

Divide the equation (A.8) by $\dot{m_{a}}$, we get a Steady-state, steady-flow (SSSF) equation for 1 kg of dry air,

$q+h_{1}=h_{2}+(\omega_{fw}-\omega_{fd})h_{w_{2}}$ (A.9)

$q+h_{a1}+ \omega_{fw}h_{v_{1}}=h_{a2}+\omega_{fd}h_{v2} +(\omega_{fw}-\omega_{fd})h_{w_{2}}$ (A.10)

$\omega_{fd}=\frac{\left[ \left( q+h_{a1}-h_{a2} \right)+\omega_{fw}\left( h_{v1}-h_{w_{2}} \right) \right]}{h_{v2}-h_{w_{2}}}$

where$h_{v}$ and *ha* denote the specific enthalpies of water vapour and dry air at DBT, respectively. $h_{w_{2}}(h_{f})$ is the specific enthalpy of water at DBT (these can be obtained from the steam table at DBT).

Enthalpy of the moist air at the inlet is calculated by using the following expression (A.11) and psychrometric chart at given DBT (Arora, 1981)

$H_{fw}=1.005T_{0}+\omega_{fw}(2500+1.88T_{0})$kJ-kg^-1^  (A.11)

The angle of incidence (*θ°*) of solar radiation on the inclined plane is calculated by the following expressions for solar angles (Duffie and Beckman, 2013):

For the azimuth angle, Eq. A.12 is used

$tan \alpha=\frac{\sin k}{\sin l\cos k-\cos l\tan d}$ (A.12)

The altitude of the sun from the normal to the surface is provided by Eq. (A.13)

$Sin \beta=\cos l\cos k\cos d+\sin l\sin d$ (A.13)

The declination angle is estimated from Eq. (A.12)

$d^{\circ}=23.4\sin\left[ \left( \frac{360\left( 284+N \right)}{365} \right) \right]$ (A.14)

The angle of incidence (θ°) for the inclined plane tilted at the angle φ° is given by,

$\cos\theta=\cos\beta\cos\alpha\cos\varphi+\sin\beta sin\varphi$ (A.15)

In this study, the azimuth angle ($\alpha)$ is taken from the south direction.

The effect of reveals height on the sunlight area (*A*_sun_) is derived through the following expression (Arora, 1981)

$A_{sun}=(L-R \sec\alpha.tan\beta)(W-R\tan\alpha)$ (A.16)

Here, *R* is the projection length.

The direct solar radiation (*I*_D_) and the diffuse radiation (*I*_d_) (Arora, 1981) are derived from Eq. (A.15) and Eq. (A.17)

$I_{D}= I_{n}\cos\theta$ (A.17)

$I_{d}=C_{s}F_{ss}I_{n}$ (A.18)

Here, *C_s_* sky radiation coefficient (Stephenson,1967), and $F_{ss}$ and $F_{sg}$ are the angle factors between the surface and sky, and the surface and ground, respectively.

The relation between the angle factors is derived by the following expression,

$F_{ss}=\left( 1-F_{sg} \right)$ (A.19)

The angle factor between the surface and ground can be deduced from Eq. (A.20)

$F_{sg}=0.5(1-sin\varphi)$ (A.20)

For the sake of comparative analysis, the geometric factor has also been included in the analysis, and it is a ratio of direct solar radiations (*I*_D_) collected on a tilted plane to that on a horizontal plane,

$$R_{D}=Cot \beta\cos\alpha cos \varphi+sin \varphi$$

The collector efficiency is derived from the following expression (Arora, 1981):

$\eta_{0}=\frac{\dot{m_{f}} \Delta H_{1}}{{A(I}_{d}+I_{D})}$ (A.21)
